# Host Phylogeny Structures the Gut Bacterial Community Within *Galerucella* Leaf Beetles

**DOI:** 10.1007/s00248-023-02251-5

**Published:** 2023-06-14

**Authors:** Yueqing An, Mariana P. Braga, Sarahi L. Garcia, Magdalena Grudzinska-Sterno, Peter A. Hambäck

**Affiliations:** 1 Department of Ecology, Environment and Plant Sciences, Stockholm University, Stockholm, Sweden; 2 Department of Ecology, Swedish University of Agricultural Sciences, Uppsala, Sweden; 3 Helsinki Life Science Institute, University of Helsinki, Helsinki, Finland; 4 Science for Life Laboratory, Stockholm, Sweden

**Keywords:** Insect-bacteria interactions, Microbial communities, Leaf beetles, Phylogenetic analysis

## Abstract

Gut microbes play important roles for their hosts. Previous studies suggest that host-microbial systems can form long-term associations over evolutionary time and the dynamic changes of the intestinal system may represent major driving forces and contribute to insect dietary diversification and speciation. Our study system includes a set of six closely related leaf beetle species (*Galerucella* spp.) and our study aims to separate the roles of host phylogeny and ecology in determining the gut microbial community and to identify eventual relationship between host insects and gut bacteria. We collected adult beetles from their respective host plants and quantified their microbial community using 16S rRNA sequencing. The results showed that the gut bacteria community composition was structured by host beetle phylogeny, where more or less host-specific gut bacteria interact with the different *Galerucella* species. For example, the endosymbiotic bacteria *Wolbachia* was found almost exclusively in *G. nymphaea* and *G. sagittariae*. Diversity indicators also suggested that α- and β-diversities of gut bacteria communities varied among host beetle species. Overall, our results suggest a phylogenetically controlled co-occurrence pattern between the six closely related *Galerucella* beetles and their gut bacteria, indicating the potential of co-evolutionary processes occurring between hosts and their gut bacterial communities.

**Supplementary Information:**

The online version contains supplementary material available at 10.1007/s00248-023-02251-5.

## Introduction

Gut microbes are important “biochemical brokers” that may enable insect herbivores to successfully exploit their host plants [1, 2]. Even though previous studies suggest that host–bacterial systems can form long-term associations over evolutionary time and represent a major driving force that contribute to insect dietary diversification and speciation [3, 4], we often lack the necessary knowledge on the microbial composition between closely related species that would enable us to identify potential coevolution between host and bacteria and to figure out how they depend on and benefit from each other. Gut microbes may be acquired through multiple sources. Insect gut microbes may be parentally inherited, where the microbes are directly transmitted from mother to progeny [5], but gut microbes may also be acquired from the environment through horizontal transmission [6]. Therefore, gut bacteria community structures could be affected both by environment factors and by host factors such as gut pH, host immune system, or nutritional condition [7–9].

Whereas determining the causes of variation among host bacterial communities is an important unanswered question in evolutionary biology, it is essentially an ecological question [10]. Ecology and evolution play important roles for the gut bacterial community structure, yet our ability to explain bacteria variation and bacteria-host relationship has remained limited. The natural world is full of examples of interactions between bacteria and their hosts, but explaining the observed variation among hosts has been difficult because insect gut bacteria may vary with a range of environmental factors, host health, host ontogeny as well as host phylogeny [11–15]. Brucker et al. [16] showed that under identical environmental conditions, the relationships of the microbial communities nevertheless reflected the phylogeny of the studied *Nasonia* host species at multiple developmental stages. This finding suggests that the structure and variation of an animal’s microbial community can be closely allied with divergence of host genes. Similarly, research on the interactions between species in the leaf beetle family *Donaciinae* and their symbionts show that symbiont-encoded pectinases evolve with reed beetles and support beetles’ folivory, and thereby also vary with the beetle phylogeny [17]. In plant sap-feeding species, it has similarly been shown that the amino acid biosynthesis evolved and interacted with their bacterial symbionts, resulting in a relationship between the bacterial community and the host phylogeny [1, 8].

One important factor affecting the spatial and temporal variation in the gut microbial community is host diet, which may reduce the predictive capacity of host phylogeny on the gut bacteria community. For instance, the gut bacterial communities in ground-dwelling beetles vary according to broad trophic habits of their hosts (carnivores, herbivores, omnivores, and scavengers) [18]. A similar variation has been observed among beetle species with similar trophic habits, such as alder leaf beetle specialists [19] and camellia weevil [20]. Because a large range of factors may affect an organism’s gut microbial community, a key issue for understanding their co-occurrence relationship with their hosts is whether gut microbiomes are inherited with their host or derived from the environment.

If the gut microbial community is inherited between life stages, then we should detect a signal from the host phylogeny on the microbial community. This question was addressed in a system including six closely related leaf beetle species (*Galerucella* spp.). The reason for selecting this set of beetle species was both extensive knowledge about their biology and a time-calibrated phylogeny [21–23]. The long-term goal is to identify key processes determining the gut microbial structure, but in this paper, we focus on the occurrence of phylogenetically controlled co-occurrence patterns in the microbial community. As a basis for the analysis, we sequenced the gut bacterial community using 16S rRNA sequencing of six beetle species, *G. lineola*, *G. tenella*, *G. pusilla*, *G. calmariensis*, *G. nymphaea*, and *G. sagittariae*, and asked how do gut bacteria communities change along the *Galerucella* phylogeny?

## Beetle Species System

**Fig. 1:**
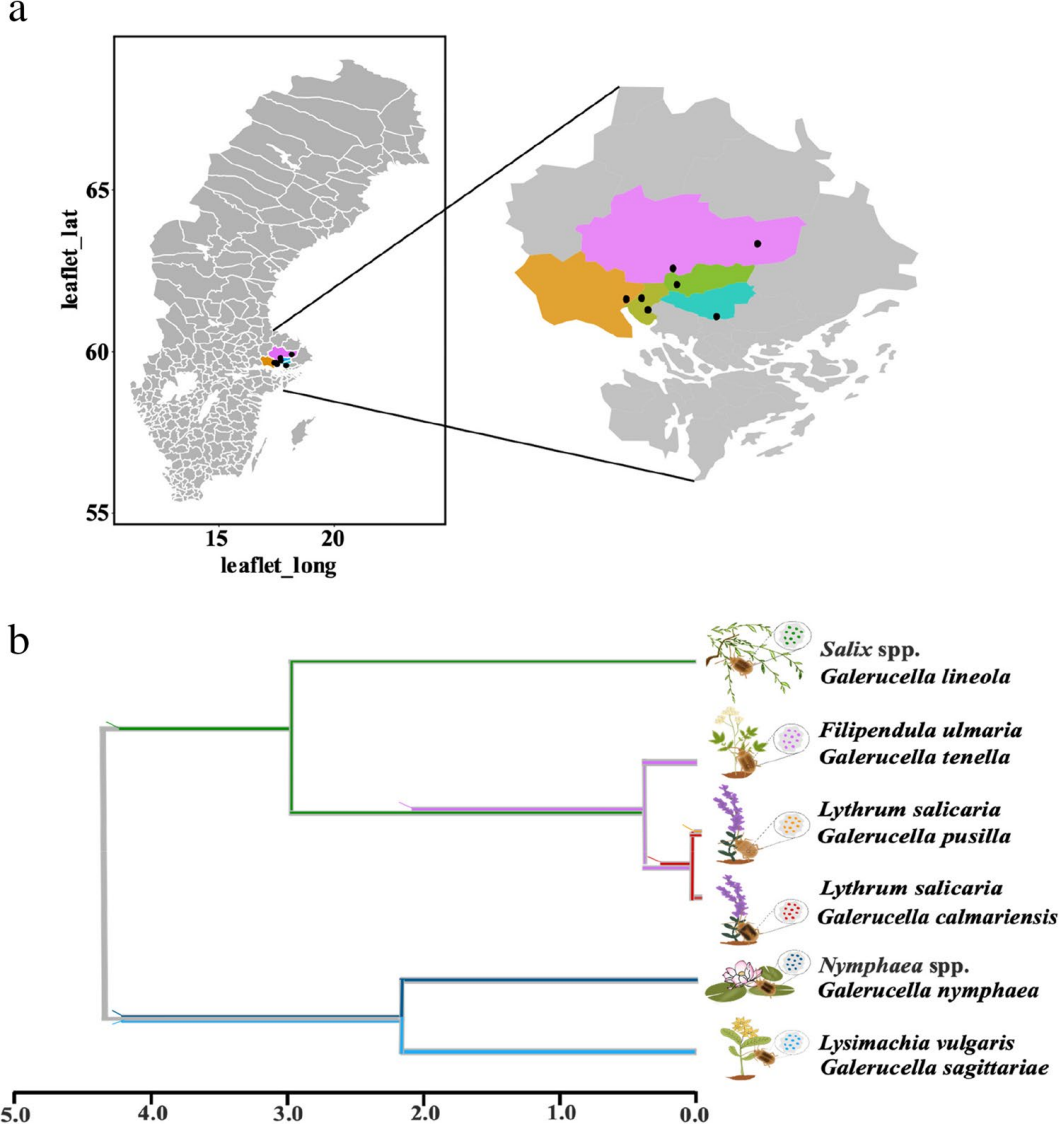
Research systems including sampling sites, beetle species, and host plant species. (**a**) Map of sampling sites in Sweden. All samples were collected from north of Stockholm (colored part in Sweden map). (**b**) Phylogenetic relationships and host plants of our six closely related study species differentiated by colors [14]. It may be that the gut microbe encodes the ability to enter the host germ line and transmit to the next generation. At the same time, under indirect inheritance, microorganisms of offspring are transmitted not only directly from their parents, but are environmentally acquired [6] (we use the same color to represent bacteria in the different *Galerucella* species in the following figures)

This study included six common *Galerucella* species, for which we have considerable previous knowledge about phylogenetic relationships for the six *Galerucella* species, including a molecular dating (Fig. 1b, [21]). This phylogeny suggested that species are differentiated by between 77 ky and 4 My, creating a wide span of relatedness. The six species have similar life cycles; overwintering as adults, egg-laying during early summer and larval development on a single host plant individual. However, one difference that may be of importance for the transfer of microbes between beetle generations concerns the fecal strings that females of four study species, often included in the subgenus *Neogalerucella*, lay on eggs (Gl—*G. lineola* (Fabricius, 1781), Gt—*G. tenella* (L., 1761), Gp—*G. pusilla* (Duftschmid, 1825), and Gc—*G. calmariensis* (L., 1767)). This fecal string is absent on eggs of the two remaining species (Gn—*G. nymphaea* (L., 1758) and Gs—*G. sagittariae* (Gyllenhal, 1813)). Another difference is that the four species with fecal strings also pupate in the ground whereas the other two species pupate on the host plant. The diet also differs between species, and four species (Gl, Gt, Gn and Gs) use multiple host plants: Gt (*Filipendula ulmaria* (L.) Maxim*.* and *Comarum palustre* L.) [24], Gl (*Alnus glutinosa* (L.) Gaertn. and *Salix* spp. L.) [25], Gs (*Lysimachia vulgaris* L., *L. thyrsiflora* L. and *C. palustre* L.) [26] and Gn (*Rumex* spp. L. and *Nymphaea* spp. L.) [27]. The only monophagous species are Gp and Gc that both have *Lythrum salicaria* L. as their only host plant.

## Methods

### Sample Collection

Adult *G. calmariensis*, *G. pusilla*, *G. tenella*, *G. nymphaea*, *G. lineola*, and *G. sagittariae* were collected from their main host plant during June, 2020 (Table 1). We collected 94 individuals in seven sites just north of Stockholm in Sweden (Fig. 1a). Individual insects were stored in 70% ethanol at 4 °C until DNA extraction.

**Table 1:** Sample information of six *Galerucella* species

Beetle species	Host plant	Sites	Geographic coordinates	# individuals
*G. calmariensis*	*L. salicaria*	Biskops Arnö	59°39′37"N, 17°28′47"E	9
		Hjälstaviken	59°39′21"N, 17°23′26"E	7
		Ekilla	59°36′19"N, 17°31′1"E	8
*G. pusilla*	*L. salicaria*	Kärven	59°54′56"N, 18°9′3"E	9
*G. tenella*	*F. ulmaria*	Nedre Föret	59°48′0"N, 17°39′42"E	7
		Kärven	59°54′56"N, 18°9′3"E	3
		Haknäs	59°43′27''N, 17°41′1''E	5
*G. lineola*	*Salix* spp.	Kärven	59°54′56"N, 18°9′3"E	7
		Hjälstaviken	59°39′21"N, 17°23′26"E	3
		Fysingen	59°34′27"N, 17°54′47"E	6
*G. sagittariae*	*L. vulgaris*	Fysingen	59°34′27"N, 17°54′47"E	7
		Kärven	59°54′56"N, 18°9′3"E	5
		Hjälstaviken	59°39′21"N, 17°23′26"E	6
*G. nymphaea*	*N. alba*	Nedre Föret	59°48′0"N, 17°39′42"E	6
		Biskops Arnö	59°39′37"N, 17°28′47"E	6

### DNA Extraction, Amplification of Microbial DNA, and High-Throughput Sequencing

We used sterile tweezers to remove the insects from the tubes with ethanol and place on a Petri dish, and thereafter removed the head, legs, wing cover, and hindwings with a sterile razor blade. The remaining body parts (thorax and abdomen) were transferred onto a filter paper and left for a few seconds to dry. After folding the filter paper and transferring the insect tissue to a clean Eppendorf tube, we stored the tubes in the fridge until extraction, which was done in the same day. The thorax and abdomen of leaf beetles were crushed for extraction. DNA extraction and purification was done using DNeasy Blood and Tissue kit (Qiagen, cat. nr 69 504). After measuring the total DNA concentration by Nanodrop, we diluted samples to 20–30 ng/μl. The V3-V4 regions of 16S rRNA sequences were then amplified using the universal primer pair 341F (5’-ACACTCTTTCCCTACACGACGCTCTTCCGATCTCCTACGGGNGGCWGCAG -3’) and 805R (5’-GTGACTGGAGTTCAGACGTGTGCTCTTCCGATCTGACTACHVGGGTATCTA ATCC -3’) attached to Illumina adapters [28]. We set up PCR reactions using KAPA HiFi HotStart Ready Mix, including 12.5 μl Kapa Hi-Fi 2x, 1.0 μl BSA (10 mg/ml), 1.0 μl Primer F (10 μM), 1.0 μl Primer R (10 μM), 7.5 μl water, and 2 μl DNA (20 ng/μl), and the following PCR protocol: (1) 98 °C for 2 min; (2) 30 cycles of 98 °C for 20 s, 49 °C for 15 s, 72 °C for 30 s; (3) 72 °C for 2 min. We included negative controls with water instead of sample DNA. PCR products were purified, quantified using Qubit dsDNA HS Assay kit, and sent to SciLifeLab for library building and sequencing on the Illumina MiSeq3 platform, including a second PCR step and two clean up steps. Cleanup based on magnetic beads MagSI-DNA NGS PREP Plus (part number MDKT00010075). Due to problems during the first sequencing run, SciLifeLab performed an additional run. Both runs were delivered to us and compared for quality and similarity. Following these procedures, we retrieved sequences from 74 and 77 samples respectively from the two runs for a total of 81 unique samples.

### Bioinformatic Analysis

Statistical analyses were conducted in R version 4.1.1 [29]. We first used the DADA2 pipeline to filter and trim raw reads [30]. The forward and reverse reads were merged to obtain the denoised sequences separately, which were clustered into an amplicon sequence variant (ASV) table. ASVs were assigned to bacterial taxa using the SILVA 138 SSU reference database [31], and the ASV table was normalized by transformation. ASVs belonging to the mitochondria family, the chloroplast order, or unassigned were removed before the downstream analysis. We used the full ASV (including singletons and doubletons) tables for proportional abundant analysis, relative abundant analysis, α-diversity analysis, but restricted the analysis to the 10 most abundant ASVs per species for proportional abundance analysis, relative abundance analysis, network analysis, and non-metric multidimensional scaling (NMDS) in order to decrease interspecies variation due to low abundant ASVs. We visualized ASVs at the beetle species level in bubble plots using ggplot2 package [32], online Venn figures (http://www.interactivenn.net) and Upset plot (https://r-graph-gallery.com) to illustrate shared ASVs among beetle species. Using the R package phyloseq [33], we calculated bacteria relative abundance per sample using abundance-based coverage estimator (ACE) and Shannon α-diversity indices. We then compared community structures between samples using NMDS with Bray–Curtis dissimilarity distances to test the role of sampling sites in structuring gut bacterial communities by visualizing dissimilarity in bacteria community composition among sampling sites for each beetle species. We also used adonis2 and pairwise adonis2, which is a wrapper function for multilevel pairwise comparison using adonis2 from package vegan, to test the role of sampling site in structuring gut bacterial communities.

We used network analysis to investigate the role of phylogeny in the cooccurrence patterns of beetle species and their bacteria communities. We used the Beckett algorithm, which breaks the network into modules and identifies the modular configuration that maximizes the proportion of interactions within modules, thus maximizing modularity [34]. Then, to test whether the identified modules have a phylogenetic component, we inferred network evolution following the approach of Braga et al. [35]. First, we inferred the evolutionary history of association between *Galerucella* beetles and their gut microbiome by modeling the potential process of gaining and losing ASVs along the beetle phylogenetic tree that produced the observed present-day interactions. For that, we included the interactions between the six *Galerucella* species and the 10 most abundant ASVs found on each beetle species (presence-absence); the *Galerucella* phylogeny of Hambäck et al. [21], and the character-based phylogenetic tree for the 45 ASVs included in this analysis. We used the model BayHost [36] as implemented in RevBayes [37]. The joint posterior distribution of model parameters and ancestral states were estimated by running three independent Markov chain Monte Carlo (MCMC) analyses for 150,000 cycles, sampling every 500 cycles and discarding the first 10% as burn-in. We used the implementation of the Gelman diagnostic [38] in the R package *coda* [39] to verify that MCMC analyses converged to the same posterior distribution. Results from a single MCMC analysis are presented. To test if the gain of an ASV was more likely when the given ASV was phylogenetically closer to the ASVs already in the beetle species, we calculated the Bayes factor comparing the prior and posterior probabilities of the parameter that controls this behavior (β) being equal to zero (following [36]). Bayes factor values smaller than 1 indicate that the bacteria phylogeny does not affect the process, values between 10 and 30 indicate strong support, and values above 100 give decisive support for the model where the bacteria phylogeny does affect the probability of gain of new bacteria [40].

Then, we used the R package *evolnets* to reconstruct ancestral networks based on the posterior probability of interaction between ancestral beetle species and each ASV. Time points were chosen based on dated species splits in the beetle phylogeny. Because the timing of the split between *G. sagittariae* and *G. nymphaea* is uncertain, we chose 2 Ma as the oldest time point. At this time point, four *Galerucella* species had diverged (*G. sagittariae*, *G. nymphaea*, *G. lineola* and *G. tenella*/*pusilla*/*calmariensis*). The next time point was 1 Ma, when *G. tenella* had diverged from the ancestor of *G. pusilla*/*calmariensis* and the final time point was the present. We discarded interactions with posterior probability lower than the threshold of 70% and used the posterior probability as interaction weight. For both ancestral networks, we identified modules the same way as we did with the present-day network, and then matched the names of the modules across networks based on the beetle species within the modules. We also performed a traditional ancestral state reconstruction (ASR), calculating interaction probabilities at internal nodes of the beetle tree.

## Results

### ASV Identification and Gut Bacterial Community Structures of Six Galerucella Species

**Fig. 2:**
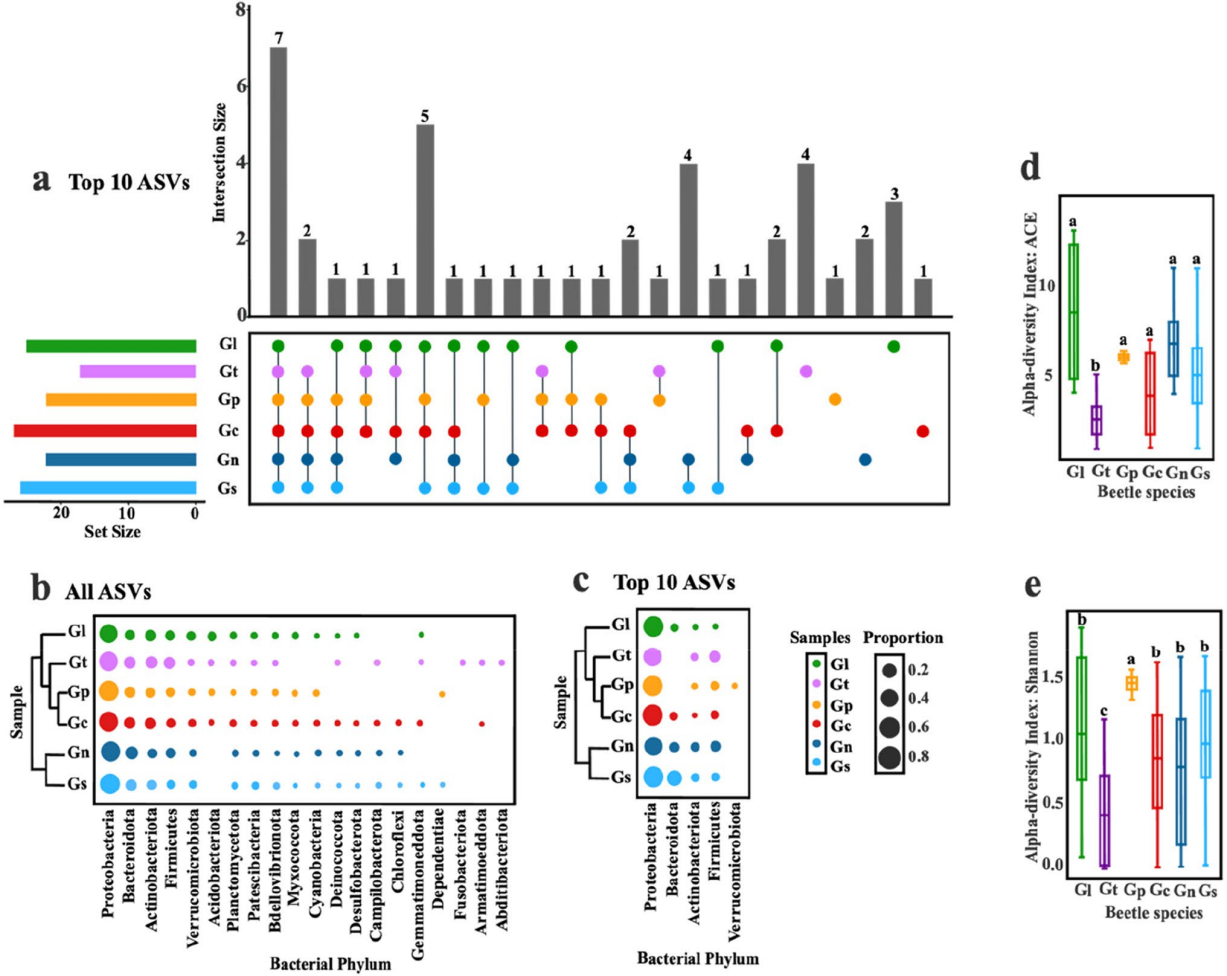
Gut bacterial structures and α-diversity of six *Galerucella* species. (**a**) Upset plot of top 10 abundant ASVs found in six *Galerucella* species. The bar plot (top) shows the number of ASVs, the matrix below the bar plot indicates which ASV are represented by each bar. The bar plots on the left show the total number of ASVs in each beetle species (coding and coloration as in Fig. 1b). (**b**) and (**c**) Proportional abundance of bacterial phyla for (**b**) all ASVs and (**c**) top 10 ASVs in six *Galerucella* species (coding and coloration as in Fig. 1b). (**d**) and (**e**) Comparison of α-diversity, (**d**) ACE indicator and (**e**) Shannon indicator of gut bacteria for each *Galerucella* species (coding and coloration as in Fig. 1b)

The rarefaction curves of ASVs in 81 samples reached an asymptote, showing that the sequencing depth is adequate. The two runs yielded almost indistinguishable ASV compositions for the same samples and were pooled before further analysis. We obtained a total of 14,157,478 raw sequence pairs from the 151 samples (7,524,314 from the first run and 6,633,164 from the second run) for a total of 81 unique samples. After quality filtering, we removed all chloroplast and mitochondria sequences, and 125 sequence variants were inferred from 12,651 input unique sequences, and clustered into 2031 unique ASVs, which means the DADA2 algorithm inferred 125 true sequence variants from the 12,651 unique sequences. All ASVs were identified and classified into 20 phyla, and the six *Galerucella* species had highly similar gut bacterial composition at the phylum level with some minor differences (Fig. 2b). α-diversity varied between *Galerucella* species (ACE and Shannon, *p* < 0.01, Fig. 2d, e) and was lower in *G. tenella* (ACE and Shannon) and higher in *G. pusilla* (Shannon). ASV numbers of *Galerucella* beetles differed between host species, the dominant phylum included Proteobacteria (57.4%), Bacteroidota (12.7%), Actinobacteriota (9.1%), and Firmicutes (6.4%) (Fig. 2b). When comparing bacteria composition between sites and within host species, adonis2 and pairwise adonis2 results showed that the top 10 gut bacteria varied between sampling site for each beetle species (Fig. S1 a-e).

### Differences of Bacterial Communities Between the Six Galerucella Beetles 

**Fig. 3:**
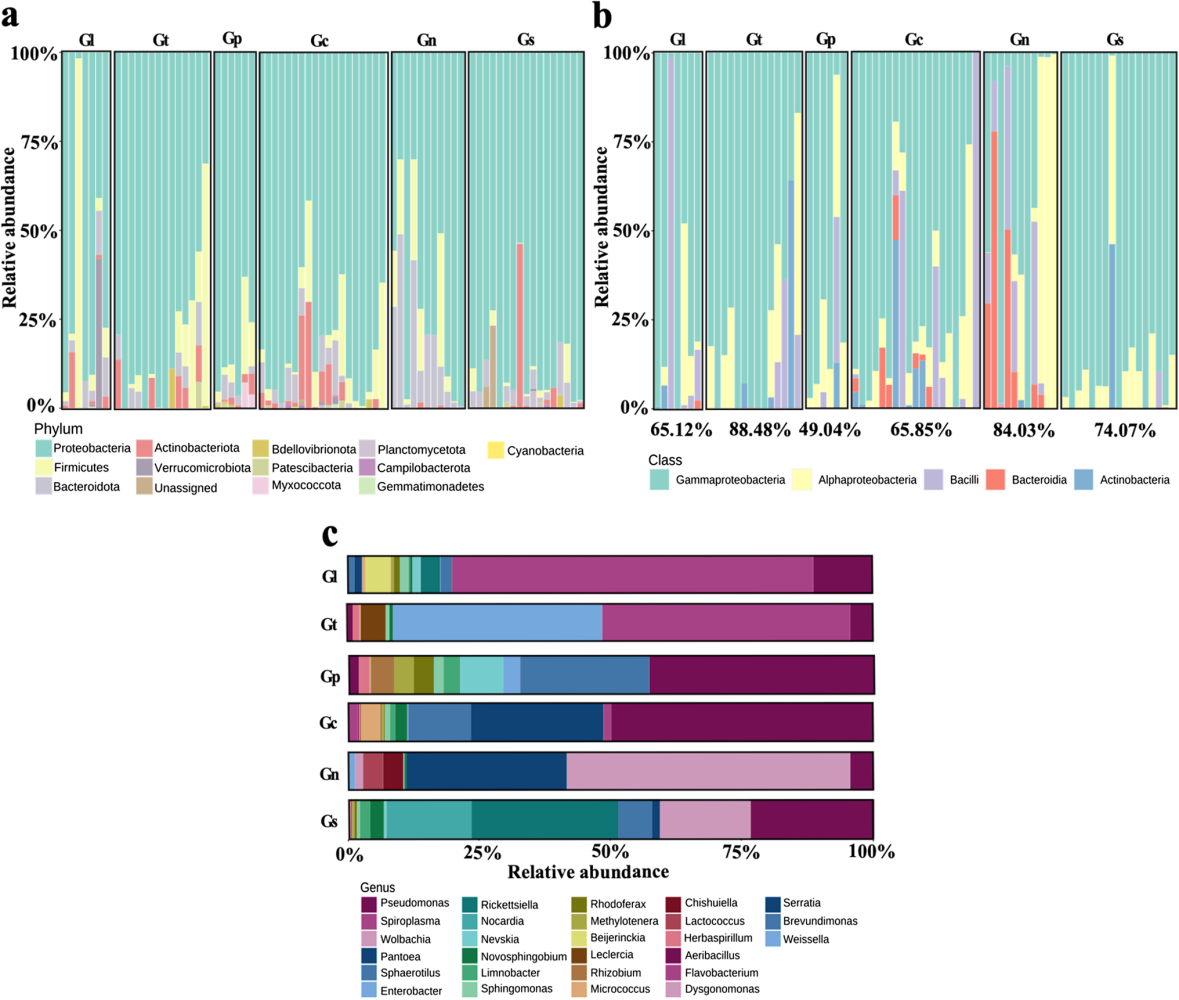
Comparisons of gut bacterial communities among different species. (**a**) and (**b**) Relative abundance of gut bacteria per sample, grouped by *Galerucella* species, (**a**) all ASVs colored at phyla level, (**b**) the 10 most abundant ASVs at class level, their proportion of total sequences in each species is under the bar chart. (**c**) Relative abundance of gut bacteria per species of the 10 most abundant ASVs at genus level (name abbreviation as in Fig. 1b)

Our analyses examining the interaction between *Galerucella* beetles and the gut bacterial communities showed that the bacterial communities were structured by the host phylogeny. This analysis included only the 10 most abundant ASVs for each beetle species, which accounted for an average of 71% of the total sequences per sample and included 45 ASVs from five phyla; Proteobacteria (*N* = 33), Bacteroidota (*N* = 3), Actinobacteriota (*N* = 2), Firmicutes (*N* = 6), and Verrucomicrobiota (*N* = 1) (Fig. 2c, for relative abundance see Fig. 3a, b). ASVs shared between the four closely related beetle species *G. calmariensis*, *G. pusilla*, *G. tenella*, and *G. lineola* increased from 1.58% when all ASVs were included to 17.78% when the set the top 10 were included, whereas the number of ASVs that were unique for a species decreased from 52.68 to 31.11% (Fig. S2).

The proportions of endosymbiotic bacteria (*Wolbachia*, *Rickettsiella*, and *Spiroplasma*) were different between beetle species (Figs. 2a and 3c). First, *Wolbachia* were common in *G. nymphaea* and *G. sagittariae* and with very low numbers in the other species. Second, *Rickettsiella* was particularly abundant in *G. sagittariae*, whereas *Spiroplasma* was abundant in *G. lineola* and *G. tenella* (Fig. 3c). Third, some genera of gut bacteria exist in all six *Galerucella* beetle species, such as *Micrococcus* (ASV39), *Novosphingobium* (ASV37 and ASV60) and *Pseudomonas* (ASV6, ASV20, ASV62, and ASV67) (Fig. 2a). Fourth, the sister species *G. nymphaea* and *G. sagittariae* share both *Wolbachia* (ASV7 and ASV48), *Nocardia* (ASV30), and *Lactococcus* (ASV42) (Fig. 2a). Finally, *Pantoea* (ASV12) and *Candidatus Rhabdochlamydia* (ASV46) only shown in *G. calmariensis* and *G. pusilla*, separately (Fig. 2a).

### Gut Bacterial Interaction with Their Host Insects

**Fig. 4:**
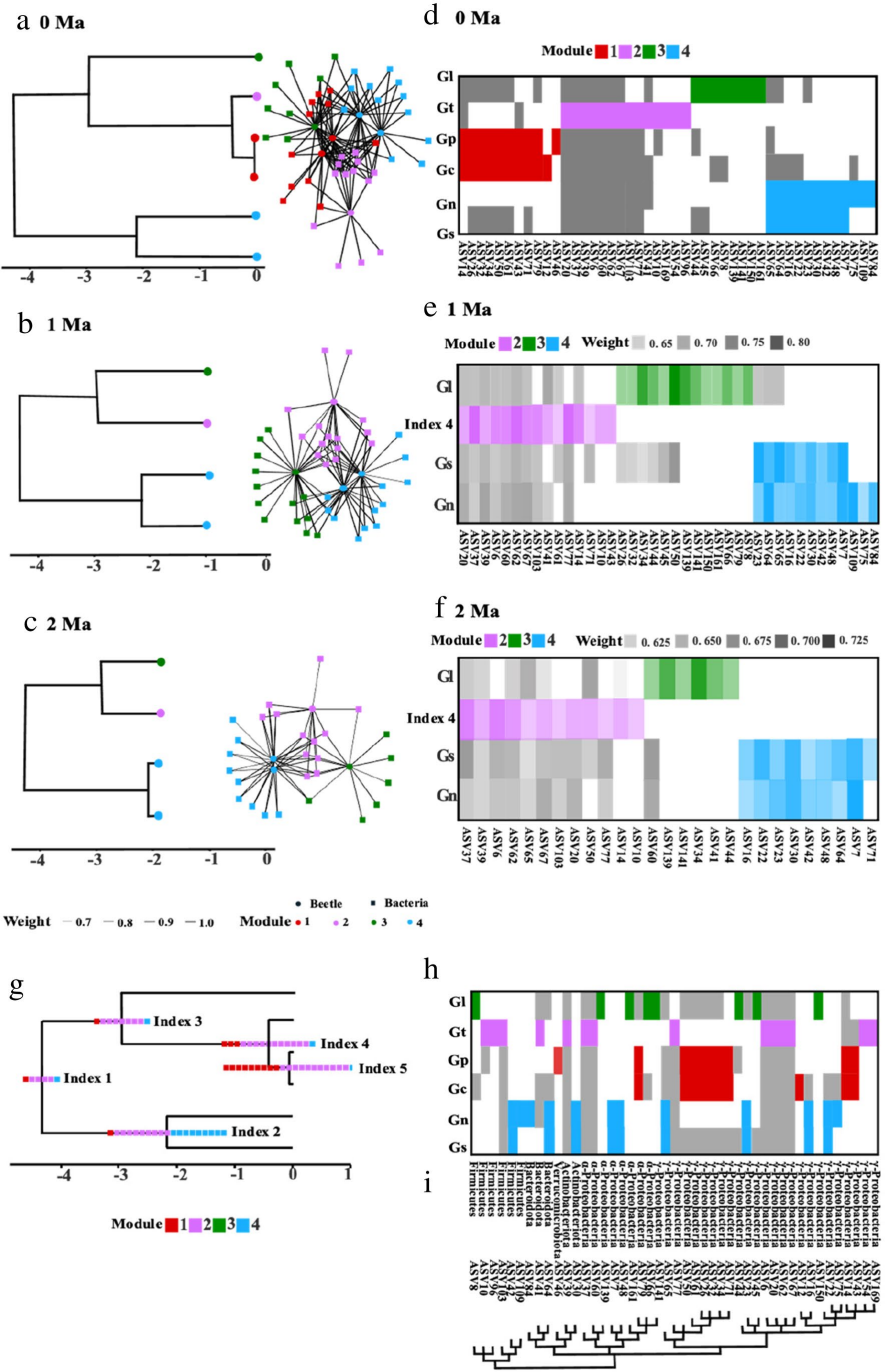
Gut bacterial interaction with their host insects. (**a**)–(**f**) Co-occurrence pattern of the top 10 abundance *Galerucella*-gut bacteria network during the past 2 Myr. (**a**–**c**) *Galerucella* phylogeny (left) and interaction network (right). (**d**–**f**) The same networks shown as matrices with rows and columns ordered by module. (**a**, **d**) show present-day observed network; (**b**, **e**) show inferred network at 1 Ma; (**c**, **f**) show inferred network at 2 Ma. For inferred ancestral networks, only interactions with posterior probability > 0.7 are shown. (**c**, **f**). Nodes in beetle phylogeny and network are colored by module in the network at the given time point. (**g**)–(**i**) ﻿Inferred ancestral interactions with gut bacteria along the *Galerucella* phylogeny. (**g**) Ancestral states with posterior probability > 0.6 at internal nodes of the host tree. (**h**) Present-day interactions colored by the module of the ASV. (**i**) Phylogenetic relationship between ASVs based on the top 10 ASVs, which belongs to Proteobacteria, Bacteroidota, Actinobacteriota, Firmicutes, and Verrucomicrobiota. Each square at the internal nodes of the host phylogenetic tree (**g**) represents one ASV and is colored by the module the ASV belongs to in the extant network (**h**)

The phylogenetic analysis identified multiple gains and losses of ASVs along the beetle phylogeny, with an average rate of 22 events per million years, split about equally between gains and losses. There was a clear phylogenetic signal in the network of interactions between beetles and their gut bacteria where closely related beetles interact with similar bacteria, and were thus placed in the same module (Fig. 4a–f). Also, model selection with Bayes factor found strong support for the model where beetles gain more easily bacteria that are closely related to bacteria already in the beetle’s microbiome (BF = 12.9). At 2 Ma, three bacterial modules were inferred: *G. lineola* in module 3, *G. nymphaea*/*sagittariae* in module 4, and *G. tenella/pusilla/calmariensis* in module 2 (Fig. 4c, f). The pattern at 1 Ma was similar to the pattern at 2 Ma, but included more specific bacteria (Fig. 4b, e). At 0 Ma, after the speciation of *G. tenella/pusilla/calmariensis*, a new module 1 (including *G. calmariensis* and *G. pusilla*) was separated from module 2 (*G. tenella*). Thus, the extant network was split in four modules, but many bacteria occurred in beetle species placed in different modules, which connects the whole network (Fig. 4a, d). The most generalist bacteria were placed in module 2, but bacteria in other modules also occurred in multiple beetle species.

The reconstruction of ancestral states at internal nodes of the beetle phylogeny shows that most of the bacteria that likely interacted with the ancestor of all six beetle species (4 out of 6) are from module 2 (Fig. 4g–i). Other bacteria in module 2 were gained as time passed across the whole phylogeny. These are common bacteria for *Galerucella* beetles, including seven Proteobacteria, one Actinobacteriota, one Bacteroidota, and one Firmicute. There are a few bacteria in module 2 that only occurred in *G. tenella*, including two Firmicutes and two Proteobacteria. Module 4 includes *G. sagittariae* and *G. nymphaea* and the bacteria that were found mainly in them include six Proteobacteria, one Firmicute, one Bacteroidota, and one Actinobacteriota. There were also three ASVs (one Proteobacteria, one Firmicute, and one Bacteroidota) that only interacted with *G. nymphaea*. Many of the bacteria in module 4 likely already interacted with the ancestor of *G. sagittariae* + *G. nymphaea,* whereas the bacteria in *G. lineola* (module 3) were most likely gained after this lineage diverged from its sister clade. Bacteria in module 1 were mainly found in the *G. pusilla* + *G. calmariensis* clade. The ancestors of *G. calmariensis* and *G. pusilla* likely interacted with 9 bacteria ASVs (all proteobacteria) from module 1 (Fig. 4g–i). *G. sagittariae* and *G. nymphaea* interact with *Wolbachia* (ASV7 and ASV48), *Spiroplasma* (ASV8, ASV96, and ASV10) existed in host-bacteria interaction patterns and interacted with *G. tenella* and *G. lineola* (Fig. 4g–i).

## Discussion

To identify differences and similarities in the microbial community structure between host species, to relate these structures to the host phylogeny, and to test whether sharing of gut bacteria is partially explained by evolutionary history, we surveyed the gut microbial community of six closely related *Galerucella* leaf beetle species. We found that the gut bacteria communities in *Galerucella* differed according to beetle species (Fig. 2 and Fig. 3) but also varied among sampling sites (Fig. S1). Our results reveal associations between the beetle phylogeny and their gut bacteria which could be an important stepping stone in explaining whether and how gut microbes have co-evolved with their hosts.

To identify whether variation of the bacteria community in *Galerucella* beetles can be explained by host phylogeny, we used stochastic mappings that were generated by a phylogenetic model of host-repertoire evolution which was then linked using network analysis to host-gut microbe co-occurrence patterns. Whereas other studies have identified core bacteriomes in relation to host phylogeny (*Dendroctonus* bark beetles and *Dryophthoridae* weevils) [4, 11], our analytical approach allows communities to be sorted along deeper branches of the phylogeny. This extension could be a further step of investigating possible co-evolutionary patterns. Coevolution between hosts and their microbiome has been shown to affect plant feeding in other chrysomelid beetles (such as Donaciinae). In those beetles, symbiotic associations with vertically transmitted bacteria provide the host beetle, that feed on pectin-rich plants, with polygalacturonases and complementing hosts’ cellulolytic enzymes [17]. In our system, we are yet unable to prove coevolutionary interactions, but the phylogenetic structuring provides some interesting candidates. First, *Wolbachia* (ASV7 and ASV48) [41, 42] was only found in the sister species *G. sagittariae* and *G. nymphaea* (Fig. 4g–i) whereas *Rickettsia* and *Spiroplasma* [43] were exclusively found in high number in some other *Galerucella* hosts.

Because the different beetle species also feed on different host plants, our results may partly confound the effect of host phylogeny and host resources, where some microbes are during feeding. Evidence showed there are not always a clear co-cladogenesis between the host and microbe communities even when gut bacteria are vertically transmitted [44], and many gut symbionts in insects are instead commensals acquired from the environment [12]. However, in *Galerucella* beetles, some patterns indicate the independent effect of host phylogeny, such as the result that the sister species *G. sagittariae* and *G. nymphaea* interact with similar bacteria despite feeding on very different host plants (Fig. 4g–i). To further understand the relationship between gut bacteria and their host insects, the next step would be to identify those gut bacteria that are transmitted between host generations, those that are connected to their host plants, and those acquired from other parts of the environment. Bruijning et al. [45] found that microbiome variation across hosts can be affected by vertical transmission fidelity, where increased fidelity reduces variation in microbial community composition across hosts whereas weak fidelity increases variation. In our results, closely related beetle species (such as *G. calmariensis* and *G. pusilla*) seemingly interact with similar bacteria, which is perhaps not surprising as they feed on the same host plant and often co-occur on the same plant individuals. In either case, one of our hypotheses is that the bacteria taxa interacting with each beetle co-evolve with the specific host species.

For the co-evolutionary hypotheses, we found some support. First, insects can maintain the stability of their symbiotic bacteria between generations. We found some bacteria taxa such as Fusobacteriota and Abditibacteriota only exist in *G. tenella*, Dependentiae only exist in *G. pusilla* and *G. sagittariae* (Fig. 2b), and for the 10 most abundant ASVs, Verrucomicrobiota only exist in *G. pusilla* (Fig. 2c). One possible explanation for this observation is that one mechanism for migration and ancestral inheritance, researchers have found that some insect species add fecal strings onto their eggs, to ensure that gut microbes are transmitted between generations. In *Galerucella*, females of some species have this behavior of adding a fecal string on the egg. The microbes can then be vertically transmitted to their offspring by female insects during egg or embryo development, or horizontally transmitted by feeding from environment and among different individuals in the same generation, so as to maintain a close relationship between the host and their symbiotic bacteria [46]. Second, microbes can promote the differentiation of the host, where immune function is an important point to discuss. The two leaf beetles *G. calmariensis* and *G. pusilla* feeding on the same host plants *Lythrum salicaria* (Lythraceae) diverged about 77 ky ago, and are currently isolated ecologically and morphologically (Fig. 1b). In order to explore the co-evolutionary relationship of gut microbes through our *Galerucella* beetle system, we need to compare transgenerational transfer between species where females do or do not add fecal strings and compare the microbial community across the life stages from adult to adult, which would be further explained in our next paper.

In conclusion, our results show that *Galerucella* species have largely species-specific bacteria that interact with each host species, suggesting that host phylogeny may act as an important genetic filter that shapes gut community structure. At the same time, geographic variation in gut communities within *Galerucella* species will create opportunities for insect hosts to partner with novel microbes that could have immune functions for their hosts.

### Supplementary Information

Below is the link to the electronic supplementary material.

Supplementary file1 (DOCX 659 KB)

## Data Availability

The datasets generated during and/or analyzed during the current study are available from the corresponding author on reasonable request.
