# Supplementary material for: Host Phylogeny Structures the Gut Bacterial Community Within Galerucella Leaf Beetles
Source: Microb Ecol. 2023 Jun 14;86(4):2477–87. doi: 10.1007/s00248-023-02251-5 (PMC10640405; doi:10.1007/s00248-023-02251-5)
Supplement: Supplementary file 1 — Supplementary file1 (DOCX 659 KB) [file 248_2023_2251_MOESM1_ESM.docx]

**Fig. S1**


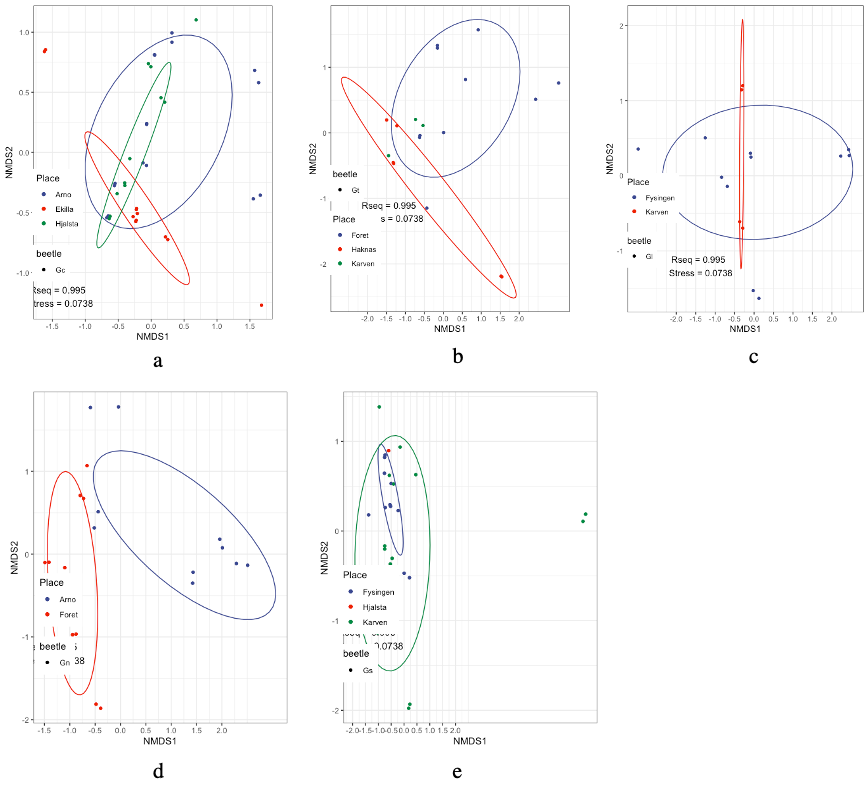


**Fig. S2
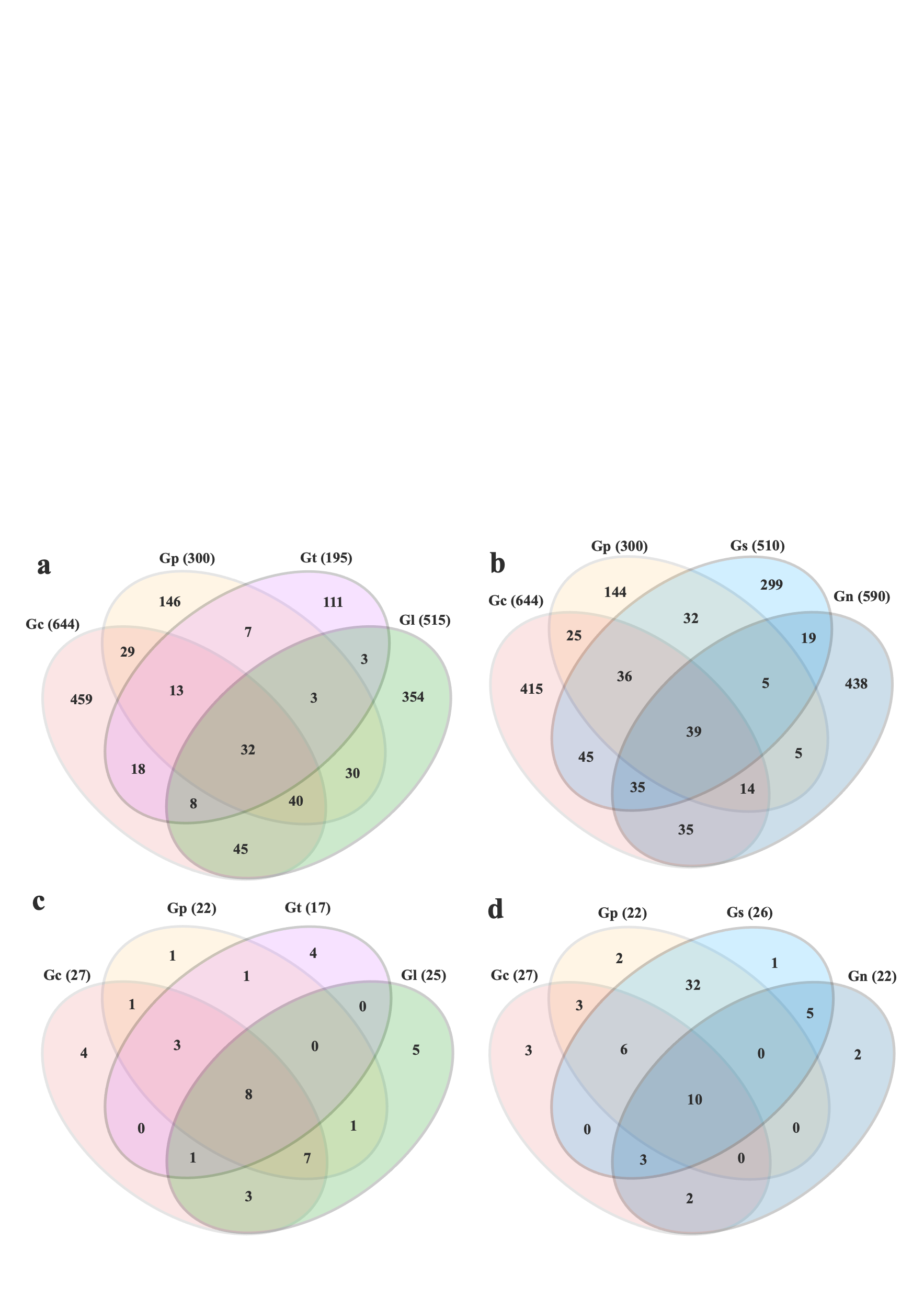
**

| **Table 2** 10 most abundant ASV table in six *Galerucella* species | | | | | | | |
| --- | --- | --- | --- | --- | --- | --- | --- |
| Taxonomy | domain | phylum | class | order | family | genus | species |
| ASV8 | Bacteria | Firmicutes | Bacilli | Entomoplasmatales | Spiroplasmataceae | Spiroplasma | Spiroplasma ixodetis |
| ASV10 | Bacteria | Firmicutes | Bacilli | Entomoplasmatales | Spiroplasmataceae | Spiroplasma | Spiroplasma ixodetis |
| ASV96 | Bacteria | Firmicutes | Bacilli | Entomoplasmatales | Spiroplasmataceae | Spiroplasma | Spiroplasma ixodetis |
| ASV103 | Bacteria | Firmicutes | Bacilli | Bacillales | Bacillaceae | Aeribacillus | Anaerobacillus isosaccharinicus |
| ASV42 | Bacteria | Firmicutes | Bacilli | Lactobacillales | Streptococcaceae | Lactococcus | Lactococcus laudensis |
| ASV109 | Bacteria | Firmicutes | Bacilli | Lactobacillales | Leuconostocaceae | Weissella | Weissella confusa |
| ASV84 | Bacteria | Bacteroidota | Bacteroidia | Bacteroidales | Dysgonomonadaceae | Dysgonomonas | Dysgonomonas macrotermitis |
| ASV41 | Bacteria | Bacteroidota | Bacteroidia | Flavobacteriales | Weeksellaceae | Chishuiella | Chishuiella changwenlii |
| ASV64 | Bacteria | Bacteroidota | Bacteroidia | Flavobacteriales | Flavobacteriaceae | Flavobacterium | Flavobacterium chungnamense |
| ASV46 | Bacteria | Verrucomicrobiota | Chlamydiae | Chlamydiales | Simkaniaceae | Candidatus Rhabdochlamydia | Candidatus Protochlamydia naegleriophila |
| ASV39 | Bacteria | Actinobacteriota | Actinobacteria | Micrococcales | Micrococcaceae | Micrococcus | Micrococcus cohnii |
| ASV30 | Bacteria | Actinobacteriota | Actinobacteria | Corynebacteriales | Nocardiaceae | Nocardia | Nocardia aurea |
| ASV66 | Bacteria | Proteobacteria | Alphaproteobacteria | Rhizobiales | Beijerinckiaceae | Beijerinckia | Beijerinckia doebereinerae |
| ASV79 | Bacteria | Proteobacteria | Alphaproteobacteria | Rhizobiales | Rhizobiaceae | Rhizobium | Rhizobium tumorigenes |
| ASV77 | Bacteria | Proteobacteria | Gammaproteobacteria | Burkholderiales | Oxalobacteraceae | Herbaspirillum | Herbaspirillum aquaticum |
| ASV50 | Bacteria | Proteobacteria | Gammaproteobacteria | Burkholderiales | Burkholderiaceae | Limnobacter | Limnobacter alexandrii |
| ASV44 | Bacteria | Proteobacteria | Gammaproteobacteria | Salinisphaerales | Sinobacteraceae; | Nevskia | Nevskia lacus |
| ASV37 | Bacteria | Proteobacteria | Alphaproteobacteria | Sphingomonadales | Sphingomonadaceae | Novosphingobium | Novosphingobium capsulatum |
| ASV161 | Bacteria | Proteobacteria | Alphaproteobacteria | Caulobacterales | Caulobacteraceae | Brevundimonas | Brevundimonas huaxiensis |
| ASV6 | Bacteria | Proteobacteria | Gammaproteobacteria | Sphingomonadales | Pseudomonadaceae | Pseudomonas | Pseudomonas spelaei |
| ASV20 | Bacteria | Proteobacteria | Gammaproteobacteria | Pseudomonadales | Pseudomonadaceae | Pseudomonas | Pseudomonas paracarnis |
| ASV62 | Bacteria | Proteobacteria | Gammaproteobacteria | Pseudomonadales | Pseudomonadaceae | Pseudomonas | Pseudomonas kairouanensis |
| ASV67 | Bacteria | Proteobacteria | Gammaproteobacteria | Pseudomonadales | Pseudomonadaceae | Pseudomonas | Pseudomonas spelaei |
| ASV23 | Bacteria | Proteobacteria | Gammaproteobacteria | Diplorickettsiales | Diplorickettsiaceae | Rickettsiella | Rickettsiella massiliensis |
| ASV45 | Bacteria | Proteobacteria | Gammaproteobacteria | Diplorickettsiales | Diplorickettsiaceae | Rickettsiella | Rickettsiella massiliensis |
| ASV26 | Bacteria | Proteobacteria | Gammaproteobacteria | Burkholderiales | Sphaerotilaceae | Sphaerotilus | Sphaerotilus montanus |
| ASV32 | Bacteria | Proteobacteria | Gammaproteobacteria | Burkholderiales | Sphaerotilaceae | Sphaerotilus | Sphaerotilus montanus |
| ASV34 | Bacteria | Proteobacteria | Gammaproteobacteria | Burkholderiales | Sphaerotilaceae | Sphaerotilus | Sphaerotilus montanus |
| ASV71 | Bacteria | Proteobacteria | Gammaproteobacteria | Burkholderiales | Sphaerotilaceae | Sphaerotilus | Sphaerotilus montanus |
| ASV60 | Bacteria | Proteobacteria | Alphaproteobacteria | Sphingomonadales | Sphingomonadaceae | Sphingomonas | Sphingomonas radiodurans |
| ASV139 | Bacteria | Proteobacteria | Alphaproteobacteria | Sphingomonadales | Sphingomonadaceae | Sphingomonas | Sphingomonas polyaromaticivorans |
| ASV141 | Bacteria | Proteobacteria | Alphaproteobacteria | Rhizobiales | Beijerinckiaceae | Beijerinckia | Beijerinckia doebereinerae |
| ASV65 | Bacteria | Proteobacteria | Gammaproteobacteria | Burkholderiales | Methylophilaceae | Methylotenera | Methylotenera versatilis |
| ASV61 | Bacteria | Proteobacteria | Gammaproteobacteria | Burkholderiales | Comamonadaceae | Rhodoferax | Rhodoferax aquaticus |
| ASV12 | Bacteria | Proteobacteria | Gammaproteobacteria | Enterobacterales | Erwiniaceae | Pantoea | Pantoea anthophila |
| ASV16 | Bacteria | Proteobacteria | Gammaproteobacteria | Enterobacterales | Erwiniaceae | Pantoea | Pantoea anthophila |
| ASV150 | Bacteria | Proteobacteria | Gammaproteobacteria | Enterobacterales | Yersiniaceae | Serratia | Serratia liquefaciens |
| ASV22 | Bacteria | Proteobacteria | Gammaproteobacteria | Enterobacterales | Erwiniaceae | Pantoea | Pantoea anthophila |
| ASV75 | Bacteria | Proteobacteria | Gammaproteobacteria | Enterobacterales | Erwiniaceae | Pantoea | Pantoea vagans |
| ASV14 | Bacteria | Proteobacteria | Gammaproteobacteria | Enterobacterales | Enterobacteriaceae | Enterobacter | Enterobacter quasihormaechei |
| ASV43 | Bacteria | Proteobacteria | Gammaproteobacteria | Enterobacterales | Enterobacteriaceae | Enterobacter | Enterobacter cloacae |
| ASV54 | Bacteria | Proteobacteria | Gammaproteobacteria | Enterobacterales | Enterobacteriaceae | Leclercia | Leclercia adecarboxylata |
| ASV169 | Bacteria | Proteobacteria | Gammaproteobacteria | Enterobacterales | Enterobacteriaceae | Enterobacter | Enterobacter quasihormaechei |
| ASV7 | Bacteria | Proteobacteria | Alphaproteobacteria | Rickettsiales | Anaplasmataceae | Wolbachia | Unassigned |
| ASV48 | Bacteria | Proteobacteria | Alphaproteobacteria | Rickettsiales | Anaplasmataceae | Wolbachia | Unassigned |
